# Wohnungleichheit in Deutschland: Erscheinungsformen, Hintergründe und Gegenmaßnahmen

**DOI:** 10.1007/s12054-021-00378-8

**Published:** 2021-04-20

**Authors:** Christoph Butterwegge

**Affiliations:** Universität zu Köln, Köln, Deutschland

**Keywords:** Wohnen, Miete, Ungleichheit, Finanzialisierung, Mietpreisgrenze, Bodenreform

## Abstract

Seit den 1980er-Jahren hat die staatliche Wohnungs‑, Wohnungsbau- und Stadtentwicklungspolitik privaten Investoren das Feld überlassen. Anschließend haben sich die Bedingungen für Mieter_innen zunehmend verschlechtert, auch weil Kapital nach der Finanzkrise bevorzugt in „Betongold“ investiert wurde. Die prekären Zustände auf dem Wohnungsmarkt für Mieter_innen sollten Anlass sein, über eine grundlegende Wende in der Wohnungspolitik nachzudenken, etwa auch in Form eines „Grundrechts auf Wohnraum“.

Mit der sich vertiefenden Kluft zwischen Arm und Reich nimmt auch die sozialräumliche Ungleichheit in der Gestalt von Segregation hierzulande deutlich zu. Einerseits ziehen sich Wohlhabende, Reiche und Hyperreiche teilweise in Luxusquartiere (Gated Communitys) hinter hohe Mauern zurück, wo sie häufig private Sicherheitsdienste bewachen; andererseits werden Arbeitslose und Arme wegen steigender Mieten vermehrt aus ihren Quartieren verdrängt. Warum ist die Ungleichheit des Wohnens entstanden und zuletzt gewachsen? Was kann man dagegen tun?

Deutlich wie nie zuvor seit Gründung der Bundesrepublik schlägt sich die Klassen- bzw. Schichtstruktur heute im Stadtbild nieder, wenn auch von lokalen Traditionen und manchen Besonderheiten gebrochen und durch andere Einflussfaktoren modifiziert. Jederzeit spürbar ist die sozialräumliche Ungleichheit in den prosperierenden Großstädten und Metropolregionen der Bundesrepublik. Der Erfurter Hochschullehrer Marcel Helbig und die WZB-Mitarbeiterin Stefanie Jähnen untersuchten die Entwicklung der residentiellen Segregation in deutschen Städten und stellten dabei fest, dass sich diese zuletzt erheblich verstärkt hat. „Ähnlich wie in den USA ist die soziale Spaltung der Städte bei Kindern bzw. Familien mit Kindern stärker ausgeprägt als bei der Gesamtbevölkerung.“ (Helbig und Jähnen 2018, S. I) Besonders ungleich verteilten sich die in Haushalten mit SGB-II-Bezug aufwachsenden Kinder. Quartiere, in denen über 50 % aller Kinder von Sozialgeld lebten, fanden Helbig und Jähnen in 36 der 74 Städte, die sie für ihre Studie ausgewählt hatten.

Wenn eine Gesellschaft wie die Bundesrepublik in sozioökonomischer Hinsicht auseinanderdriftet (vgl. hierzu: Butterwegge 2020, 2021), gehört ihr sozialräumlicher Zerfall zu den brisantesten Folgen. Da sich Ungleichheit keineswegs darauf beschränkt, dass die Gesellschaftsmitglieder unterschiedlich viel besitzen oder unterschiedlich hohe Einkommen haben, sondern fast in sämtlichen Lebensbereichen deutliche Spuren hinterlässt, ist auch das Wohnen betroffen – heute vielleicht *die* Soziale Frage in Deutschland schlechthin.

Durch die Bundesrepublik verläuft ein tiefer Riss, der sie in ein gesellschaftliches Oben und Unten sowie in wohlhabende und abgehängte Regionen, Kommunen und Stadtviertel teilt. Zu beobachten ist außerdem, was man eine sozioökonomische Sezessionsbewegung nennen kann: Während die Einkommensschwachen, Geringverdiener_innen und Transferleistungsbezieher_innen abgehängt und in die Hochhausviertel am Rand der Großstädte abgedrängt werden, weichen die materiell Bessergestellten in gute und separate Wohnviertel bis hin zu Gated Communitys aus. Sie ziehen sich aus freien Stücken in eine Parallelwelt zurück, die Privilegierten vorbehalten bleibt, und der eine ganz andere Welt gegenübersteht, die nicht selbstgewählt ist und der Unterprivilegierte nur schwer entfliehen können.

Von der „Herstellung gleichwertiger Lebensverhältnisse“, die Art. 72 Abs. 2 *Grundgesetz* fordert, kann selbst mehr als drei Jahrzehnte nach der Vereinigung von BRD und DDR noch keine Rede sein. Ein sozialräumlicher Ausgleich, wie ihn dieser „politische Leitbegriff“ vorsieht, der laut Jens Kersten, Claudia Neu und Berthold Vogel (2019, S. 4) ein „Verfassungsauftrag für öffentliches Handeln“ ist, hat bisher nicht stattgefunden: „Daseinsvorsorge und Infrastrukturen stehen nicht überall in angemessenem Umfang zur Verfügung, um die gesellschaftliche Teilhabe aller Bürgerinnen und Bürger zu gewährleisten. Dies gilt nicht nur für den ländlichen Raum, sondern auch für viele großstädtische Quartiere, die unter Segregation leiden.“

In den wirtschaftlich erfolgreichen Ballungszentren greifen vermehrt Wohnungsnot und Mietwucher um sich, weshalb es zumindest in den meisten Groß- und Universitätsstädten selbst Normalverdiener_innen schwerfällt, eine bezahlbare Wohnung zu finden. Verschärft wird das Problem durch eine sehr niedrige Wohneigentumsquote der Bundesrepublik, die so niedrig ist wie in keinem anderen Land der Eurozone (vgl. European Central Bank 2020, S. 1), was mit der extrem starken Konzentration des Vermögens zusammenhängt. In den Städten wohnen die allermeisten Menschen zur Miete, weil die Wohnungen entweder reichen Privatleuten oder großen Immobilienunternehmen gehören, denen es häufig in erster Linie um eine hohe Rendite geht.

## Ursachen der wachsenden Ungleichheit im Wohnbereich

Da die Einkommens- und erst recht die Vermögensverteilung viel ungleicher ist als die Verteilung der Miethöhen, finden Personen, Familien und Haushalte mit geringem Einkommen oft keine für sie bezahlbaren Wohnungen. „Deshalb verschärft der Wohnungsmarkt schon durch seine Preisstruktur die Einkommensungleichheit.“ (Lohauß 2019, S. 310) Über die Lage, Größe und Ausstattung der Wohnung bzw. des Eigenheims entscheidet die finanzielle Leistungsfähigkeit, was dazu führt, dass die Spaltung der urbanen Quartierswelt voranschreitet.

Die gegenwärtige Wohnungsmisere und der „Mietenwahnsinn“ sind aber nicht vom Himmel gefallen, sondern durch politische Entscheidungen erzeugt worden. Seit den 1980er-Jahren überließ die staatliche Wohnungs‑, Wohnungsbau- und Stadtentwicklungspolitik privaten Investoren das Feld. CDU, CSU und FDP schafften zum 1. Januar 1990 das *Wohngemeinnützigkeitsgesetz* ab. Damit hatte der Staat z. B. genossenschaftlichen Wohnungsbaugesellschaften bis Ende der 1980er-Jahre bestimmte Steuervorteile gewährt, sie dafür jedoch zur Beschränkung auf eine Kostenmiete und zur Begrenzung von Gewinnausschüttungen verpflichtet. Vorher preisgebundene Wohnungsbestände gelangten daraufhin auf den Immobilienmarkt, wo es primär um hohe Renditen ging. „Mit dem Fortfall der Gemeinnützigkeit wurden die öffentlichen Wohnungsgesellschaften zum Beutegut von kapitalistischen Immobilienunternehmen, die als sogenannte Heuschrecken die Wohnungsbestände aufkauften.“ (Krüger 2017, S. 225)

Mit vier Finanzmarktförderungsgesetzen schufen unterschiedlich zusammengesetzte Bundesregierungen seit 1990 ein günstiges Investitionsklima und ein ideales Betätigungsfeld für (institutionelle) Kapitalanleger, nicht zuletzt im Bereich der Immobilien (vgl. Heeg 2018, S. 107 ff.). Mietwohnungen, die eine Mehrheit der Bevölkerung benötigt, um hierzulande menschenwürdig leben zu können, werden seither mit der Folge als Waren ge- und als bloße Spekulationsobjekte behandelt. Hingegen leidet der Soziale Wohnungsbau unter Schwindsucht, weil ihn die politisch Verantwortlichen nicht mehr vorantrieben: Gab es zur Jahrtausendwende über 2,5 Mio. öffentlich geförderte Wohneinheiten, waren es nach Angaben der Bundesregierung am 31. Dezember 2019 bloß noch 1,14 Mio.. Außerdem wurde das Mietrecht liberalisiert und der in Deutschland für Vermieter traditionell relativ strenge Kündigungsschutz gelockert. Mit der sog. Modernisierungsumlage von elf Prozent führten SPD und Bündnis 90/Die Grünen zum 1. September 2001 eine Beteiligung der Mieter an den Modernisierungskosten ein, und die am 1. Mai 2013 in Kraft getretene Mietrechtsreform der schwarz-gelben Koalition erleichterte Zwangsräumungen.

Die rot-grüne Koalition befreite Gewinne von Kapitalgesellschaften, die aus dem Verkauf von Tochterfirmen und Aktienpaketen anderer Kapitalgesellschaften resultierten, von der Körperschaftsteuer – eines der größten Steuergeschenke an die Unternehmen überhaupt. „Daraufhin stießen etliche Unternehmen ihre Beteiligungen ab, welche in der Folge unter anderem von Private-Equity-Fonds gekauft wurden. Auch die Käufer von privatisierten Wohnanlagen waren meist Private-Equity-Fonds.“ (Metzger 2020, S. 131) Folglich war die hierzulande später als in den angelsächsischen Staaten einsetzende Finanzialisierung des Immobilienmarktes kein Rückzug des Staates aus diesem Bereich, sondern ein rasanter Aufstieg besonders kapitalkräftiger Eigentümer, der von Parlament, Regierung und Verwaltung unterstützt wurde.

Philipp P. Metzger (2020, S. 228) hat die Finanzialisierung des Mietwohnungsmarktes in der Bundesrepublik mit jener in den USA verglichen und auf einen wesentlichen Unterschied hingewiesen: Während sie in den Vereinigten Staaten eine stärker individualisierte Form angenommen und zu einer „Eigentümernation privat verschuldeter Haushalte“ geführt habe, sei die Finanzialisierung in Deutschland auf eine Mieternation getroffen, weshalb wenige große Unternehmen als Hauptakteure dominiert hätten, ohne dass sich Privathaushalte maßlos überschulden mussten.

Seit dem 1. Januar 2004 sind auch in Deutschland die in den USA kurz nach dem Zweiten Weltkrieg entstandenen Hedgefonds, seit dem 1. Januar 2007 auch die sog. REITs (Real Estate Investment Trusts) gesetzlich zugelassen, welche in den USA bereits 1960 eingeführt wurden. Dabei handelt es sich um steuerbegünstigte Immobilien-Aktiengesellschaften, denen zwar der Kauf von Bestandsimmobilien verboten war, durch deren Geschäftsmodell sich der Privatisierungsdruck aber weiter erhöhte. Obwohl stadtentwicklungs-, sozial- und demokratiepolitische Argumente, die Sebastian Klus (2020, S. 89) rekapituliert, gegen eine Privatisierung öffentlicher Wohnungsbestände sprachen, haben der Bund, aber auch manche Länder und viele Kommunen, dem neoliberalen Zeitgeist gehorchend, teilweise ihren gesamten Wohnungsbestand – häufig sogar zu Schleuderpreisen – an US-amerikanische Investmentgesellschaften, internationale Finanzinvestoren und börsennotierte Immobilienkonzerne verkauft. Dadurch beraubten sich die Gebietskörperschaften auf Jahrzehnte selbst der Möglichkeit, eine zielgerichtete Stadtentwicklungspolitik zu machen und vor allem die Wohnungsversorgung einkommensschwacher Bevölkerungsgruppen zu sichern.

## Folgen der Privatisierung öffentlicher Wohnungsbestände und der Finanzialisierung des Mietwohnungsmarkts

Seit 2015 gehört der von „Deutsche Annington“ in „Vonovia“ umbenannte Immobilienriese zu den 30 wertvollsten Konzernen, die im Dax gelistet sind. Seine exorbitanten Profite erwirtschaftete das Unternehmen durch Luxussanierungen und Wertsteigerungen seines wachsenden Immobilienbestandes, rüde Methoden der „Entmietung“ und gesetzlich erlaubte Mieterhöhungen von bis zu elf bzw. acht Prozent nach Modernisierungsmaßnahmen. Auch die Deutsche Wohnen, der vor allem in der Bundeshauptstadt und im Rhein/Main-Gebiet viele Mietshäuser gehören, hat es nach Gewinneinbußen der Lufthansa während der Covid-19-Pandemie in den Dax geschafft. Seither drohen ihren Mieter_innen aufgrund der hohen Renditeerwartungen ausländischer Investoren und großer Vermögensverwalter einerseits noch rigidere Praktiken des Immobilienkonzerns. Andererseits hat sich die Konzernpitze aufgrund des Negativimages, des Volksbegehrens „Deutsche Wohnen & Co. enteignen!“ und des im Januar 2020 vom Berliner Abgeordnetenhaus beschlossenen Mietendeckels offenbar für einen weniger rücksichtslosen Umgang mit den Mieter_innen entschieden.

Auf dem deutschen Mietwohnungsmarkt haben Wohnimmobilien-AGs zuletzt erheblich an Bedeutung gewonnen. Sie erreichen zwar nur einen Marktanteil von etwa vier Prozent, dieser konzentriert sich jedoch auf die lukrativen Metropolregionen, weil Konzerne wie Deutsche Wohnen, LEG und Vonovia nicht in der Fläche präsent sind (vgl. Metzger 2020, S. 194). Wie der Kölner Publizist Werner Rügemer (2020, S. 50) konstatiert, haben Kapitalorganisatoren wie BlackRock & Co. als Eigentümer und Vermieter großer Wohnungskomplexe maßgeblich zur Mietenexplosion in deutschen Städten beigetragen. Nicht die Wohnungsmärkte sind also implodiert, sondern Konzerne – durch politische Weichenstellungen dazu motiviert – auf diesen lukrativen Wirtschaftssektor expandiert. Finanzinvestoren haben diesen für die ganze Bevölkerung existenzwichtigen Lebensbereich noch stärker ihrer Profitlogik unterworfen.

Die meisten Kapitalanleger fürchteten nach der globalen Finanz‑, Weltwirtschafts- und europäischen Währungskrise 2007/08 ff. weitere Bankpleiten und Börsenzusammenbrüche, weshalb „Betongold“ in der Folgezeit immer beliebter wurde. Aufgrund des Immobilienbooms nach dieser Krisenerfahrung hat sich die sozioökonomische Ungleichheit in Deutschland verschärft. Da sich das Immobilieneigentum bei Wohlhabenden, Reichen und Hyperreichen (Hochvermögenden) konzentriert, haben die Wertsteigerungen bei Häusern und Wohnungen erheblich zur Vertiefung der Kluft zwischen Arm und Reich beigetragen.

Nach den Immobilienpreisen stiegen keineswegs nur in bevorzugten Stadtlagen auch die Mieten für Normal- und Geringverdiener_innen. Längst müssen viele Haushalte einen Großteil ihres Einkommens für Mietzahlungen aufwenden, was ihnen nur einen geringen Spielraum für Anschaffungen und andere notwendige Ausgaben lässt. Mieter_innen wurden gewissermaßen enteignet, weil sie in dieser Phase extrem niedriger Hypothekenzinsen keine adäquaten Einkommenszuwächse verzeichneten. „Wenn Miet- und Bodenpreise (trotz billigen Geldes) steigen, wird das Einkommen lohnabhängiger Mieterinnen und Mieter in Kapital- und Bodenvermögen transferiert. Das heißt, dass sich die Enteignung der Mieter insofern verschärft, als eine Umverteilung von unten nach oben stattfindet.“ (Hubeli 2020, S. 100)

Gleichzeitig sinkt die Zahl der Sozialwohnungen, die einer Mietpreis- und Belegungsbindung unterliegen, seit Jahrzehnten. Andrej Holm (2014, S. 163) bemängelt allerdings zu Recht, dass Sozialwohnungen in Deutschland nur für eine gewisse Zeit der Mietpreis- und Belegungsbindung unterliegen. Er hält den sozialen Wohnungsbau für ein „zutiefst ineffektives Förderinstrument“, weil das Zweifache des Baupreises an die finanzierenden Banken einerseits und die die Eigentümer zwecks Sicherung der Eigenkapitalverzinsung andererseits gezahlt werde: „Die öffentliche Hand zahlt doppelt für etwas, das schon nach wenigen Jahren einem Privatmann oder einer Investitionsgesellschaft gehört.“

## Gegenmaßnahmen von einer Bodenreform über die Mietpreisbremse bis zum öffentlichen Wohnungsbau

Raumordnungs‑, Stadtentwicklungs- und Wohnungspolitik dürfen nicht an den Kapitalverwertungsinteressen von (Groß‑)Investoren, sondern müssen an den Bedürfnissen der (potenziellen) Bewohner_innen von Stadtteilen orientiert sein. Ertragserwartungen von Finanzinvestoren und Wohnbedürfnisse von Mieter_innen sind weitgehend unvereinbar. Nirgendwo versagt das kapitalistische Wirtschaftssystem so eklatant wie bei der Wohnungsversorgung. Da sich der Markt als unfähig erwiesen hat, eine adäquate Wohnungsversorgung für alle Bevölkerungsschichten sicherzustellen, muss sie als öffentliche Aufgabe begriffen und vom Staat aus Gründen der sozialen Verantwortung für seine Bürger_innen gewährleistet werden, dass niemand wegen seines geringen Vermögens und seines zu niedrigen Einkommens auf der Strecke bleibt. Statt die „immobilienwirtschaftliche Landnahme“ deutscher Städte zu fördern, müssen Parlamente, Regierungen und Verwaltungen laut Andrej Holm (2014, S. 171 f.) den „Ausstieg aus einer profitorientierten Wohnungspolitik“ vollziehen. Dies fällt den politisch Verantwortlichen immer schwerer, weil die Macht der Immobilienwirtschaft und der großen Wohnungsunternehmen ständig wächst.

Der ehemalige Münchner Oberbürgermeister und spätere Bundesminister für Raumordnung, Bauwesen und Städtebau, Hans-Jochen Vogel, hat bis zu seinem Tod im Juli 2020 immer wieder die zentrale Bedeutung des Bodens und der Bodenpreise hervorgehoben. Da es sich beim Grund und Boden um eine „Grundvoraussetzung menschlicher Existenz“ handle, die man nicht „dem unübersehbaren Spiel der Marktkräfte“ überlassen könne, müsse hier politisch angesetzt werden, wolle man das Problem der Preisexplosion im Wohnungsbereich lösen, meinte Vogel (Vogel 2019, S. 48): „Die Wertschätzung des knappen und unentbehrlichen Gutes Boden darf sich nicht länger in spekulativen Gewinnerwartungen ausdrücken, sollte vielmehr im Sinne einer nachhaltigen und gemeinwohlorientierten Nutzung erfolgen, die den Boden als wesentliche Grundlage der Daseinsvorsorge sowohl für die heutige Bevölkerung als auch für die kommenden Generationen anerkennt.“

Folgerichtig schlug der SPD-Politiker eine Kommunalisierung vor, d. h. die Überführung wohnbaurelevanter Grundstücke in Gemeindeeigentum. „Sie scheiden damit aus den Markt-Gütern aus, deren Preis mit der Absicht des Wertzuwachses und des Vermögensgewinnes nach den Regeln von Angebot und Nachfrage in die Höhe getrieben werden. Denn ihre Wertsteigerung kommt nur noch den Gemeinden zugute.“ (Vogel 2019, S. 71) Zu einer neuen und gerechten Bodenordnung gehörte für Vogel, dass eine Gemeinde wohnungsrelevantes Eigentum nicht mehr an Privatleute verkauft, sondern bloß noch in Erbpacht vergibt, damit sie die Kontrolle über den Boden behält.

Ergänzend befürwortete Vogel (2019, S. 33) die Einführung eines Planungswertausgleichs, was er mit „ganz elementaren Gerechtigkeitserwägungen“ begründete: „Es kann nicht angehen, dass Bodeneigentümer für jeden öffentlichen Eingriff Entschädigung erhalten, die Gewinne, die ihnen durch öffentliche Entscheidung, also beispielsweise durch die Zuerkennung von Baurecht, erwachsen, aber für sich behalten können.“ Mit einer Bodenwertzuwachssteuer könnte man die Spekulation mit Grundstücken weniger lukrativ machen und leistungslose Gewinne abschöpfen. Durch erweiterte Satzungsbefugnisse würden die Kommunen in die Lage versetzt, die Stadtentwicklung effektiver im Sinne des Gemeinwohls zu mitzugestalten.

Mit einer halbherzigen „Mietpreisbremse“ für Teilwohnungsmärkte, die CDU, CSU und SPD zum 1. Juni 2015 eingeführt, aufgrund unbefriedigender Erfahrungen mit diesem Instrument zweimal „nachgeschärft“ und gleichzeitig bis zum 31. Dezember 2025 verlängert haben, ist das Problem des Wohnungsmangels für Einkommensschwache jedenfalls nicht zu lösen. Die mancherorts geradezu skandalösen Zustände auf dem Mietwohnungsmarkt sollten vielmehr Anlass sein, über eine grundlegende Wende in der Wohnungspolitik nachzudenken.

Da sich Räumungsklagen und Zwangsräumungen ausgerechnet in den Großstädten mit ihren angespannten Mietwohnungsmärkten seit geraumer Zeit mehren (vgl. Holm 2014, S. 121), ist die Verankerung eines „Grundrechts auf Wohnraum“ in unserer Verfassung überfällig, für das der spätere Außenminister und Bundespräsident Frank-Walter Steinmeier zu Beginn der 1990er-Jahre in seiner juristischen Dissertation *Das polizeiliche Regime in den Randzonen sozialer Sicherung. Eine rechtswissenschaftliche Untersuchung über Tradition und Perspektiven zur Verhinderung und Beseitigung von Obdachlosigkeit* plädiert hat. Staat und Behörden müssten, forderte Steinmeier (1992, S. 394 f.), per Grundgesetzauftrag „zum Bau und Erhalt preisgünstigen Wohnraums für breite Bevölkerungskreise“ verpflichtet werden, und es dürfe keine Wohnung z. B. wegen aufgelaufener Mietschulden geräumt werden, bevor nicht „zumutbarer Ersatzwohnraum“ zur Verfügung stehe.

Zweckmäßiger als eine Wiederbelebung des sozialen Wohnungsbaus in der überkommenen Form wären

- die Ausweitung des öffentlichen Wohnungsbaus, sinnvollerweise ergänzt durch eine soziale Mietpreisgestaltung, sowie
- eine Wiederherstellung der Wohnungsgemeinnützigkeit, um die Aktivitäten genossenschaftlicher und kommunaler Wohnungsbaugesellschaften staatlicherseits zu stimulieren.

Würden sie dazu finanziell in die Lage versetzt, könnten deutsche Kommunen dem Vorbild der österreichischen Hauptstadt („Rotes Wien“) nacheifern und auch durch eigene Bautätigkeit mehr Wohnungen für auf einem preisbildenden Markt eher Chancenlose schaffen. Außerdem müssen die längst bestehenden Gestaltungsspielräume der kommunalen Wohnungspolitik konsequenter genutzt werden. Beispielsweise kann die Umwandlung von Miet- in Eigentumswohnungen durch Soziale Erhaltungs- bzw. Milieuschutzsatzungen und Nutzung des kommunalen Vorkaufsrechtes ebenso erschwert werden wie die Verdrängung einkommensschwacher Bevölkerungsschichten aus den Innenstädten mittels Luxusmodernisierungen.
